# Acceptability to Healthcare Professionals of Home-Based HPV Self-Sampling for Cervical Screening: A French Qualitative Study Conducted in an Area with Low Access to Health Services

**DOI:** 10.3390/cancers15215163

**Published:** 2023-10-26

**Authors:** Johane Le Goff, Anne-Sophie Le Duc-Banaszuk, Caroline Lefeuvre, Adeline Pivert, Alexandra Ducancelle, Hélène De Pauw, Marc Arbyn, Aubeline Vinay, Franck Rexand-Galais

**Affiliations:** 1 University of Angers, CLIPSY, SFR CONFLUENCES, F-49000 Angers, France; aubeline.vinay@univ-angers.fr (A.V.); franck.rexand-galais@univ-angers.fr (F.R.-G.); 2 Pays de la Loire Regional Cancer Screening Coordination Center (CRCDC Pays de La Loire), F-49000 Angers, France; as.banaszuk@depistagecancers.fr; 3 University of Angers, CHU Angers, HIFIH, SFR ICAT, F-49000 Angers, France; caroline.lefeuvre@chu-angers.fr (C.L.); adpivert@chu-angers.fr (A.P.); alducancelle@chu-angers.fr (A.D.); 4 Cancer Epidemiology Unit, Belgian Cancer Center, Sciensano, 1050 Brussels, Belgium; helene.depauw@sciensano.be (H.D.P.); marc.arbyn@sciensano.be (M.A.); 5 Department of Human Structure and Repair, Faculty of Medicine and Health Sciences, Ghent University, 9000 Ghent, Belgium

**Keywords:** cervical cancer, cancer screening strategies, underscreened women, low physician density, urine self-sampling, vaginal self-sampling, health professionals, semi-structured interview

## Abstract

**Simple Summary:**

Cervical cancer is an important and preventable disease. Logistical, financial, organizational, and psychological barriers must be addressed to reach adult women not attending screening. The present qualitative study examined these barriers for women and explored effective screening strategies in areas of France with fewer doctors and suboptimal access to health facilities. Healthcare professionals were interviewed on the advantages and disadvantages of mailing vaginal or urine self-sampling kits. Self-sampling has been shown to be an effective tool for reaching underscreened women. Our survey highlighted several items that need to be considered in future screening programs that will include self-sampling: (1) development of clear user instructions, (2) choice of an effective and acceptable self-sampling device (vaginal or urine), (3) involvement of health professionals, and (4) support for women (information about the sequence of events (collection, results communication, and follow-up of screen-positive patients)).

**Abstract:**

Self-sampling may improve participation in cervical cancer secondary prevention programs by women who do not respond or respond irregularly when invited to contact a health professional for the collection of a cervical specimen. It could also help resolve access problems in areas with a low physician density. The present qualitative study examined barriers to screening, effective screening strategies, and the advantages and disadvantages of sending women urine or vaginal self-sampling kits in two medically underserved administrative departments in France (Mayenne and Sarthe) showing low cervical screening coverage. As part of the CapU4 randomized trial, a team of psychologists investigated the attitudes and experiences of 59 healthcare professionals (gynecologists, general practitioners, and midwives) through semi-structured interviews. Results indicated that health professionals believe that self-sampling may address the issues of low physician density and underscreening by removing logistical, organizational, financial, and psychological obstacles. They confirmed trust in the use of vaginal self-sampling, with urine self-sampling as an alternative solution (e.g., for women with vaginismus). The health professionals also identified several limitations of the self-sampling kit that will need to be addressed in future screening campaigns (incomplete kit, complex instructions, poor anatomical knowledge, and obesity).

## 1. Introduction

Cervical cancer (CC) is the second most common cause of death by cancer in women under the age of 45 years in developed and industrialized countries worldwide [1]. CC is, however, preventable via the vaccination of teenagers against the human papillomavirus (HPV) and secondary prevention comprising HPV screening every 5 to 10 years from age 30 years. HPV-based screening provides 60–70% greater protection than cytology for invasive cancers [2].

Systematic reviews have demonstrated that patient self-sampling provides accuracies similar to those of clinician-sampling for HPV testing and, moreover, represents an opportunity to reach underscreened women [3]. Women appear to appreciate and better accept self-sampling [4], preferring it over clinician-sampling [5]. However, only 17 countries (including France) with screening programs currently recommend its use, and only eight recommend it as a means for reaching underscreened populations [6], despite the fact that the World Health Organization describes it as a simple and relevant approach [7]. Sending out self-sampling kits appears to be more effective for reaching underscreened women than invitations or reply letters to contact a healthcare worker for cervical sampling [3,8]. The French National Authority for Health (Haute Autorité de Santé [HAS]) has recommended vaginal self-sampling (VSS) as an alternative to clinician-taken samples since 2019 and specifies that this self-sampling should be offered to unscreened or insufficiently screened women starting at 30 years of age [9]. In France, there is no organized distribution of VSS kits. However, women can collect a VSS from a medical biology laboratory if they know about it. Also, a medical biology laboratory proposed VSS to health professionals. However, this is not achieved throughout the territory.

HPV testing can also be performed on first-void urine and might be more acceptable than VSS [10]. Many studies have demonstrated the effectiveness of urinary self-sampling (USS) for increasing adherence to CC screening. Its effectiveness in detecting precancerous and cancerous lesions of the cervix has also been demonstrated [11]. The use of HPV tests on urine collected with the Colli-Pee device has been clinically evaluated and validated in the VALHUDES study [12]. Colli-Pee is a device that collects only the first-void fraction and mixes the collected urine with a conserving liquid. Two published studies showed good performance of three HPV tests on Colli-Pee collected urine versus on clinician-collected samples [13,14]. One of the three evaluated assays is the BD Onclarity HPV Assay, which is the test used in the CapU4 trial [15]. However, USS is currently only proposed in research protocols [16], and thus, it is not currently offered as a screening alternative.

The French national cervical screening program recommends HPV testing once every five years for women aged 30–65 years [17] and aims to achieve a coverage rate of 70% by making screening accessible to vulnerable populations and/or people living in areas with poor healthcare provision. In the Pays de la Loire administrative region, cervical screening coverage was 62.3% in the 2018–2020 period. There were considerable disparities between its administrative departments, with the mainly rural Mayenne and Sarthe departments having the lowest rates (respectively 60.5% and 58.3%) [18]. The national screening program also aims to improve screening practices and optimize those of relevant healthcare professionals. In France, gynecologists, general practitioners (GPs), and midwives are the main providers of screening [19]. Their role is to inform women about screening, deliver clear and objective information on the challenges of screening to them, and offer testing [19]. The National Cancer Institute (INCa) emphasizes the need to systematically inform women and ask them about their last screening test. However, many areas in Pays de la Loire have low physician density, and some are considered to be medical deserts. Sarthe and Mayenne [20] have the lowest physician density. Although healthcare professionals play a major role, little is known about their opinions on cervical screening and self-sampling. Several studies have highlighted reticence among them as concerning the performance of self-sampling and the risk of missing opportunities to address other health issues [21,22,23].

The CapU4 study aims to offer an alternative strategy, vaginal or urinary self-sampling at home, to women who do not regularly participate in CC screening. This study, therefore, aims to explore the viewpoints of health professionals with regard to cervical screening in priority intervention areas (PIAs) on an alternative system that could be implemented in the future to improve screening coverage.

## 2. Materials and Methods

The CapU4 research project, whose methodology is described elsewhere [15], compares responses to invitational USS and VSS strategies to the current procedure involving traditional reminder letters to increase participation in cervical screening. The present qualitative part of CapU4 investigated screening attendance obstacles and means to surmount them, as well as the opinions of primary healthcare professionals on self-sampling. More specifically, the objective was to understand the different situations those professionals encounter, the resources they use, the difficulties they face, and any remaining points potentially able to improve both the system and its methods (e.g., sending the self-sampling kit to the women’s homes versus to their care providers). The GPs, midwives, and gynecologists we interviewed worked in Mayenne or Sarthe (Pays de la Loire, France), two rural administrative departments with low physician density and low screening coverage.

The present study was based on the analysis of 59 semi-structured interviews carried out between April and August 2022. The CapU4 research project was reviewed and approved by the Sud-Est I institutional review board (2021-123, 25 November 2021, France). The French Data Protection Authority was informed about the use of recordings for the study in September 2021 (ref. 2223607v0).

### 2.1. Semi-Structured Interviews

Based on theoretical elements and previously explored practices [21,22,23,24], the psychologists within the CLiPsy team in the Department of Psychology at Angers University (France) established guidelines for the interviews of healthcare professionals. These guidelines contained questions addressing general and profession-specific topics such as screening practices, how they are discussed and proposed, barriers to screening attendance, opinions, knowledge, attitudes regarding self-sampling, and propositions that may facilitate screening in PIAs. The interview guidelines can be found in the Appendixes: (Appendix A, p. 12) for gynecologists, (Appendix B, p. 13) for GPs, and (Appendix C, p. 14) for midwives.

### 2.2. Population

Healthcare professionals working in rural, semi-rural areas or in the PIAs of Mayenne or Sarthe (Pays de la Loire, France) were selected. Five professional criteria, such as age, gender, years of practice, geographical area of practice, and mean number of CCs encountered in the previous two years, were collected. Interviews were conducted until response saturation was reached [25].

### 2.3. Process and Analyses

Healthcare professionals had previously been informed by e-mail of the study’s objectives and conditions by the Pays de la Loire Regional Cancer Screening Coordination Center (CRCDC). This nonprofit public health body provides organized colorectal, breast, and cervical cancer screening. For the present work, the CRCDC furnished contact details for a requested 733 healthcare professionals, specifically 589 GPs, 40 gynecologists, and 104 midwives. E-mails inviting the professionals to participate and explaining the objectives of the research were then sent. For GPs and gynecologists, 13 reminder e-mails were sent at a pace of once per week. For midwives, five reminder e-mails were sent. We asked candidate participants to suggest a time when they would be available for a videoconference or telephone conversation. We explained that the study was confidential and voluntary. All interviews were conducted by a CLiPsy team psychologist, recorded with the participant’s consent, and anonymized.

The interviews were analyzed using standard qualitative content analysis [26]. Grounded theory [27] was used as the benchmark for this qualitative approach. We applied the constant comparison method between emerging and previous data. The interviews were subjected to a thematic analysis, whereby the answers of each participant were summarized to reveal the main themes for each profession. This allowed us to compare opinions across professions, determine similarities and differences in the data, and identify the characteristics and relationships between the different components of the processes [28,29]. The initial interviews for each of the professionals were always supplemented by two additional interviews to confirm findings and achieve data saturation.

## 3. Results

### 3.1. Participants

The sample comprised 59 healthcare professionals, among whom were 14 gynecologists, 25 GPs, and 20 midwives (i.e., successful recruitments of 14/40 for gynecologists, 25/589 for GPs, and 20/104 for midwives). Equipment failure hampered the recording of two of the 59 interviews, but the notes taken during the interview enabled their inclusion in the analysis. Interviews lasted 11 to 40 min. The sociodemographic characteristics of the sample are set out below in Table 1.

### 3.2. Barriers to Cervical Screening

**Figure 1:**
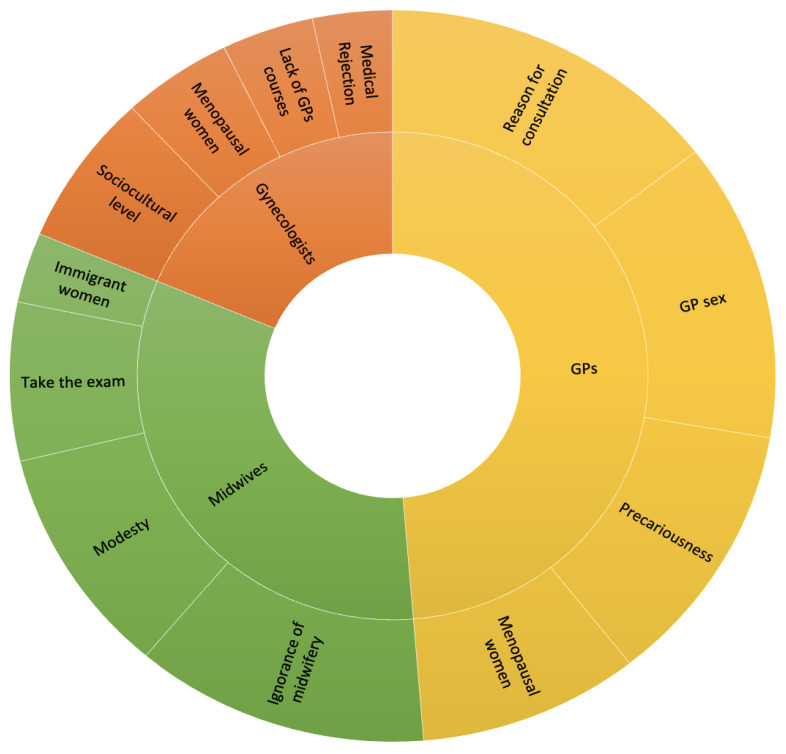
Barriers to cervical screening according to healthcare professionals.

Given the PIA context, our analysis revealed several barriers to screening according to professional category. We selected four factors per profession, following conventional qualitative content analysis [26]. Figure 1 presents these factors. Some factors were common, but others differed between professions.

The interviewed healthcare professionals agreed with the organization of the screening program. More specifically, they acknowledged the usefulness of follow-up letters, reminding women of the deadlines for screening and encouraging them to consult. Nevertheless, this seemed inadequate, especially in PIAs, where the main problem is geographical and linked to the problem of accessing GP care.

#### 3.2.1. Gynecologists

Gynecologists reported usually examining women who have understood the importance of cervical screening and attend regularly. They thus rarely encountered non-attenders, with comments such as “They come because they want to be screened” (Gyn6), for example. The gynecologists mentioned immigrant status as an important barrier to screening, e.g., “Screening is certainly not a priority for immigrant populations” (Gyn13). Sociocultural aspects appeared as another obstacle, particularly with regard to understanding information: “We need to communicate better based on socio-cultural level” (Gyn 2); “These are patients who do not have the medical culture we have in more urban areas” (Gyn6). The influence of the socio-economic level was also noted, as well as the isolation of populations with economic precariousness: “In rural areas, health is not always the priority” (Gyn1). Postmenopausal women, who may confuse screening with gynecological follow-up, appeared to contact gynecologists only rarely: “Patients between 50 and 60 years old, at the end of their professional activity, with grown-up children—their last gynecological examination was at the birth of their last child” (Gyn1). Gynecologists tended to assume that GPs play a central role in cervical screening, although they sometimes flagged a lack of training: “GPs and other members of the medical profession are not well trained” (Gyn9); “The professionals on the front line are the GPs, but GPs are not trained enough in screening” (Gyn6). Finally, gynecologists identified rejection of medical services as another barrier to cervical screening, linked to the medical violence encountered by some patients: “There is a suspicion of mistreatment by gynecologists, a rejection of medical care. It is a state of mind popping up due to events in the United States (…) There is abuse, that’s for sure, but it’s not widespread.” (Gyn9).

#### 3.2.2. General Practitioners

GPs underlined that they did not focus exclusively on gynecology, stating, for example, that “There are other things in general medicine besides cervical cancer” (GP9) or “There’s a distinction to be made between prevention, emergencies, and medical follow-up” (GP24). In addition, not all GPs performed gynecological consultations, with only 16 of the 25 interviewed GPs offering cervical screening. This was the first barrier our respondents identified in their profession, explaining that the many different reasons for consulting a GP sometimes made it impossible to focus on screening: “We’re very busy with other issues; prevention takes second place” (GP10). Respondents also identified a barrier that affected patients and healthcare professionals alike, namely the GP’s gender: “There are women who don’t want to be examined by a man” (GP22); “I am a man, which isn’t great at the moment in gynecological medicine” (GP9). From a social point of view, GPs also identified deprivation as a factor reducing the priority of screening: “In deprived environments it’s complicated” (GP7); “More difficult with a low social level, and a low level of education too” (GP15); “Taking the sample was complicated because of the language barrier” (GP19); “It’s more the cultural and educational level that influences screening” (GP15). Finally, menopause was identified by GPs as an obstacle to cervical screening: “Menopausal women who think that it’s over, and they no longer need it” (GP1); “Menopausal women don’t always see the benefit of screening” (GP7); “At menopause, they feel less concerned” (GP12).

#### 3.2.3. Midwives

Midwives also had specificities but shared some aspects with gynecologists, mentioning notably that “If women come to see a midwife, it means that they agree to the smear” (MID3). The midwife respondents believed that ignorance of their profession partially accounted for the low coverage of cervical screening: “Several times a week, I hear people expressing surprise at the fact that midwives screen for it” (MID5); “To further highlight the skills of midwives” (MID2). According to them, that aspect was the first obstacle to cervical screening. They also highlighted a psychological factor, specifically shyness, which they believed was a primary factor for not attending screening. They cited women’s relationships with their bodies: “For women who have problems with their intimacy, I think it’s a real obstacle” (MID2); “We must overcome shyness” (MID11). Midwives also identified the content of the consultation as a possible hindrance, including the technical and psychological aspects: “Women who have concerns with their intimacy or a traumatic history” (MID2); “As soon as they see the word ‘cancer’, some people throw the invitation to get tested in the bin” (MID7); “fitting the speculum can be complicated” (MID19). Finally, immigration was discussed. Some of the midwives did not encounter this population but thought that it must be a key factor for screening non-attendance. Immigrant status was depicted as a real obstacle to screening, preventing it from being performed or even mentioned: “Immigrants who are monitored are seen more at local mother and child protection centers than in consultation or at the hospital” (MID15); “I am thinking of asylum-seekers, there is the language barrier. There are other priorities before thinking about screening”; “It is most often the upper classes who are screened and who have follow-up” (MID16).

Each profession identified specific barriers to cervical screening. These differences were linked to the missions of the health professionals, the patients they see, and their patients’ representations.

### 3.3. Barriers to Self-Sampling According to Healthcare Professionals

**Figure 2:**
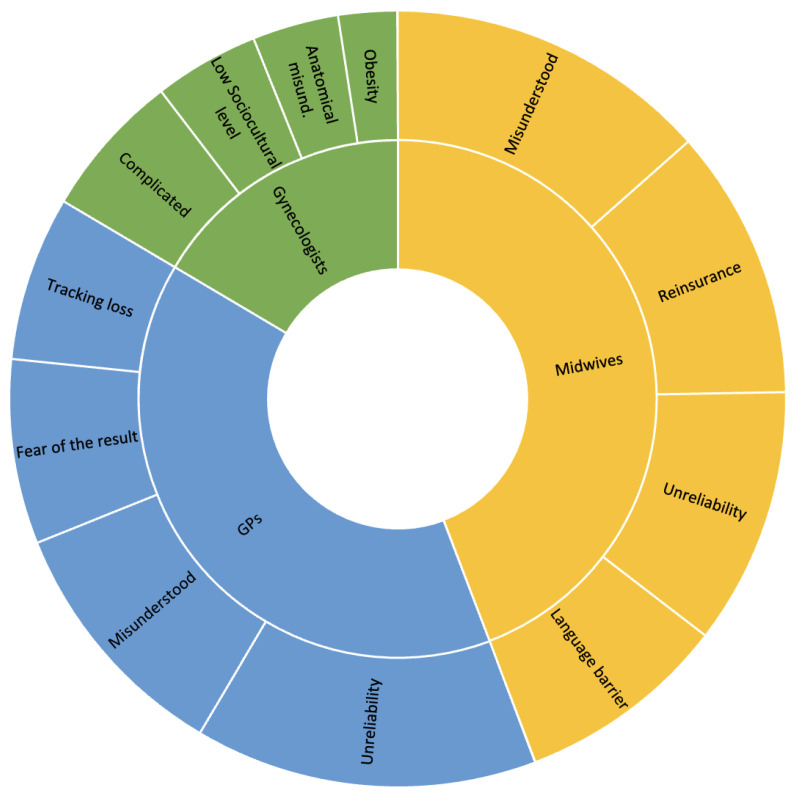
Barriers to self-sampling identified by healthcare professionals.

Figure 2 displays the barriers regarding self-sampling highlighted by the three professional groups.

Notably, healthcare professionals stated that self-sampling should not replace the comprehensive consultation that takes place during cervical screening.

#### 3.3.1. Gynecologists

The interviewed gynecologists were concerned whether women would understand how to use the kits. They found the kits complicated, particularly the one for USS, making such comments as “Vaginal self-sampling is clear, urine self-sampling is not. It’s not clear what the woman is supposed to do” (Gyn4). They also questioned the choice of organ: “The urinary tract is more complicated … whereas putting something inside the vagina is more explicit. Intellectually, it’s easier to understand” (Gyn1). More specifically, they worried about a lack of anatomical knowledge: “Women in their 60s, who are still concerned by cervical screening, just don’t have that knowledge of their anatomy” (Gyn4); “A patient put the swab in her urethra” (Gyn6).

Illiteracy was also mentioned as something that could interfere with self-sampling by limiting a woman’s ability to understand and use the kit. Some professionals suggested ways of improving this aspect: “Pictures, illustrated instructions (…) Modeled on the labs that produce instructions for the contraceptive pill (…) they do comic strips now (…) No reading, something more fun” (Gyn1). From an anatomical point of view, respondents felt that self-sampling would be difficult for obese women: “It’s a technical obstacle. I’m not sure they’d be able to reach their cervix” (Gyn6).

#### 3.3.2. General Practitioners

GPs also wondered whether women would understand the kit, mentioning that “They come more because they haven’t understood the kit” (GP9) or “It’s really reserved for people who understand what it’s all about” (GP14). These physicians, therefore, questioned its effectiveness and reliability: “Self-sampling is easier to do but there is a loss of reliability” (GP10). This lack of confidence was especially marked for USS: “It might be better to do the vaginal one, I’d have more confidence” (GP5); “I have some concerns about the quality of the urine sample” (P12); “I don’t understand how we can trust urine screening for cancer, when we’re talking about the cervix” (GP24). The existence of USS for CC screening was a surprise for many of the GPs, with only 6/25 of them aware of its effectiveness in that setting.

Losing touch with their patients was also a worry for GPs: “I’m afraid they’ll say to themselves that they’re good to go for the next five years, but the gynecological consultation is also important” (GP3); “It has to remain a one-off, complementary (…) we have to continue to see them” (GP5). Furthermore, they believed that women would be afraid of the results of self-sampling: “Be present to reassure women and take over when HPV is positive” (GP2). They felt that the self-testing kit should be accompanied by appropriate medical information: “information from the general practitioner, teaching, explaining why, explaining what we gain from it” (GP10); “communicate on the usefulness (…) on how we do it” (GP12).

#### 3.3.3. Midwives

The midwives agreed with the other professions on the difficulty of understanding and using the kit, commenting that “They came with their kit because they didn’t understand it” (MID4) and “If the women are intelligent, it’s OK, but if they have mental deficiencies, it would be more complicated” (MID20). Similarly, like GPs, midwives believed that patient support was needed to allay any fears caused when self-testing kits are sent directly to patients’ homes. They recognized the importance of reassurance: “The information is important, but you have to take the time to explain it” (MID4); “They don’t fully read the instructions in the explanatory note (…) they’re afraid of doing something wrong” (MID5); “We need to reassure, support self-sampling” (MID9). In addition, midwives wondered about the reliability of self-sampling in terms of both its completeness and its effectiveness: “I’m afraid they’ll skip the general consultation” (MID1); “I’d be afraid of it being less reliable” (MID16); “I’d tend to say that the vaginal self-sampling is a little more reliable” (MID6). Finally, the midwives identified the language barrier as hindering the use of the self-sampling kit: “For asylum-seekers, there’s a real language barrier”; “Clearly if there’s a language barrier, it’s impossible to understand how to use it” (MID16).

### 3.4. Benefits of Self-Sampling

The self-sampling facilitators are shown below in Table 2. All health professionals agreed on these facilitators.

#### Specificities Regarding Urine and Vaginal Self-Samplings

GPs and midwives thought that VSS was more relevant than USS for detecting HPV, stating, for example, “I have some doubts about the urine self-sampling” (GP14), “I would be afraid of it being less reliable” (MID16) or “Technically, urine testing is more complex to explain and understand” (MID4). VSS was also perceived as closer to the conventional method for detecting sexually transmitted infections in routine practice: “We are more used to prescribing vaginal tests” (GP2). Gynecologists generally viewed VSS and USS as having equivalent reliability but nonetheless questioned the relevance of sending USS kits to women: “They’re so simple I’m afraid some people won’t use them” (Gyn11); “I put myself in their shoes: if I received one, I’d ask myself what a urine kit is doing in screening for cervical cancer” (Gyn13).

USS was preferred for its ease of use, though, given that women are more accustomed to taking urine samples than cervical samples. These kits also seemed more appropriate for some populations (e.g., women with vaginismus or a history of sexual abuse): “For difficult populations like those with vaginismus, vaginal self-sampling can be difficult” (Gyn9); “it would be more relevant for women who find gynecological examinations an ordeal, such as women with vaginismus” (GP5); “urinary self-sampling is suitable for those with vaginismus (…) it’s less intrusive” (MID4).

Performing VSS also requires knowledge of one’s anatomy, which could be a limitation, according to health professionals: “Women must already know their bodies” (MID5). In addition, health professionals questioned the use of VSS for two categories of women. First, for those over 50, they highlighted the possibility of pain due to vaginal dryness when taking the sample: “Menopausal women without mucus, we won’t have much HPV” (Gyn9). Second, for those with morbid obesity, who might find it difficult to take the sample: “You have to be comfortable with your body to perform this sampling” (GP14).

## 4. Discussion

We focused our qualitative analysis on barriers to cervical screening and barriers and facilitators to self-sampling by interviewing primary healthcare professionals in a PIA. The obstacles to cervical screening identified by our respondents were in line with and complementary to a recent report by INCa [30]. For example, being a man was clearly a barrier for the healthcare professionals in our study, as it was in previous work [31]. Sociocultural level, deprivation, and the language barrier were also identified as obstacles to cervical screening, confirming the 2022 findings of Chandrakumar et al. [32]. The citation of obesity as both a technical and psychological barrier by our healthcare providers was consistent with the 2022 study by Urbute et al., wherein obesity was associated with lower participation in cervical screening [33].

Other factors included the role of healthcare professionals in screening and particularly a lack of knowledge on the subject. Some respondents were not aware of the recommendations for screening or of the possibility of HPV self-sampling, particularly USS (e.g., 6/25 of GPs). Indeed, this strategy is little known by health professionals because it is not yet validated in France as an alternative strategy to screening. It is only proposed in research protocols [16]. However, not all professions can implement these recommendations, given the shortage of physicians [20] and especially GPs. Time constraints, administrative burdens, and low availability—given the many other reasons for consulting—interfere with the ability to offer and perform screening [34].

In addition, screening must remain a fully supported system, especially in isolated and rural areas, as emphasized in another INCa study [35]. According to healthcare professionals, sending out invitation letters for testing appears to increase the participation rate, but this may not be enough, especially in rural areas and PIAs. Indeed, when preceded by a doctor’s call or a face-to-face meeting to explain their use, the participation rate associated with self-sampling kits increases [8]. Also, results from several studies suggest that sending self-sampling kits to the homes of unscreened women is more effective and more cost-effective than just relying on follow-up letters [36,37]. Another alternative might be to send invitation letters to nonadherent women while at the same time sending GPs a list of their nonadherent patients [38].

The obstacles to self-sampling identified by healthcare professionals can be categorized into two groups: those related to the kit itself and those related to its use. Respondents described the self-sampling kits as incomplete and complex. They described them as incomplete because self-sampling does not replace gynecological consultation. It is not to keep women away from the healthcare system. This does not exempt women from consulting a health professional to have an annual gynecological examination (breast examination, cervix observation, contraception, etc.). Furthermore, healthcare professionals suggested simplifying the kits, making their use as agreeable as possible, and focusing on making them easier to understand for people facing a language barrier [39]. Our respondents emphasized that the higher the level of illiteracy, the more necessary it becomes to explain why the kit is important and how it works using a simple and appropriate vocabulary. According to the French National Illiteracy Agency (ANLCI), 7% of the adult population in mainland France is illiterate, and half of them live in rural or sparsely populated areas [40]. Pictures or illustrated instructions would be better to ensure a good understanding in this context. However, device instructions do need to conform to regulatory standards. For example, the USS instructions meet the CE declaration of conformity. Thus, adapting them to the needs of specific sub-populations may prove challenging for the companies offering self-sampling devices.

Our respondents also pointed out that in order to use a self-sampling kit, women must have sound knowledge of their anatomy, and most of them (38/59) doubted that they did. To address this issue, the healthcare professionals suggested CC awareness campaigns to reach as many women as possible. Awareness was also a major point among Reunion Island healthcare professionals belonging to the RESISTE research program when they assessed the acceptability of home self-sampling [24].

Although our healthcare professionals were not unanimous when it came to the reliability of the kits, they did acknowledge that self-sampling is appropriate for hard-to-reach populations, in line with HAS guidelines [9] and Wood et al. (2018) [41]. More specifically, USS raised questions. Only six of our 25 interviewed GPs were aware of its use to detect HPV, and its reliability was questioned despite its effectiveness [10,11,12,13,14]. Indeed, considering reliability, most of our healthcare professionals (42/59) expressed a preference for VSS. Nonetheless, they did believe that USS would be better for certain women, especially those who lack anatomical knowledge, suffer from vaginismus, have a history of sexual abuse, or present obesity. USS is surely easier for women to perform, given the probability that most have already self-collected urine samples, whereas few have self-collected cervical samples.

In general, the healthcare professionals we surveyed viewed self-screening as acceptable insofar as it does not require speculum use, often cited by healthcare professionals as intrusive for women [42,43]. The kits also solve the problem of people who reject all things medical as a result of medical violence they may have endured in the past [44]. Of the three professions we surveyed here, GPs were the most favorable for the implementation of self-sampling, a finding consistent with those of Zelli et al. (2022) [23].

Via our qualitative study, we are able to suggest recommendations that may contribute to improving CC screening participation among women living in disadvantaged areas of France. These recommendations are presented below in Box 1.

Box 1 **Recommendations to improve cervical cancer screening attendance in France formulated by health professionals, derived from the CapU4 interview.**

– Continue sending out invitation letters as part of the routine screening program;
– Provide information for the public: television campaigns, articles in women’s magazines;
– Pursue face-to-face meetings with women (nursing homes, depist-bus mobile screening unit);
– Make the kits free of charge;
– Continue to inform and train healthcare professionals in the use of these kits;
– Raise GP awareness of cervical screening;
– Include easily understandable user instructions in the sent self-sampling kits;
– Offer USS to specific populations;
– Reassure women about their ability to carry out self-sampling;
– Involve healthcare professionals when conceiving strategies including self-sampling: raising awareness, informing women (the accompanying leaflet will sometimes have to be explained by a healthcare professional or translated);
– Reassure women about the availability of a professional if necessary;
– Improve links to healthcare professionals (who should be consulted when the HPV test result is positive);
– Make it clear that self-sampling does not exempt women from consulting a health professional to have an annual gynecological examination (breast examination, cervix observation, contraception, etc.)

## 5. Conclusions

Cervical cancer is a public health problem. It is the second most common cause of death by cancer in women under the age of 45 years in developed and industrialized countries worldwide. Prevention remains the cornerstone for reducing cancer mortality. It is thus crucial to identify effective preventive measures.

Our qualitative study was conducted under the aegis of the CapU4 research project and was one of the first to study the attitudes of healthcare professionals toward self-sampling screening. Moreover, it assessed the acceptability and effectiveness of sending out home self-sampling kits as a means of reaching underscreened women. In France, organized screening for CC uses several facilitators aimed at improving participation, such as reminder letters sent to non-responding women. However, the program seems insufficient, especially for underscreened women in PIAs. Sending out home self-sampling kits appears to be an acceptable strategy to reach these women. The use of self-sampling kits may mitigate logistical, financial, organizational, and psychological (fear, embarrassment, etc.) barriers. Offering self-sampling to women who have not been screened for more than four years may be an appropriate strategy for meeting the World Health Organization’s objective of eliminating CC. It is a strategy that healthcare professionals consider acceptable for PIAs.

## Figures and Tables

**Table 1:** Sociodemographic characteristics of participating healthcare professionals.

		Sex		Working Area				
	Age	Women	Men	Rural	Urban	Rural + Urban	Years of Medical Practice	Cancer Cases
Gynecologists *n* = 14	48	11	3	/	11	3	18	3
	Min = 32; max = 75							
GPs *n* = 25	44	13	12	18	5	2	18	1
	Min = 28; max = 70							
Midwives *n* = 20	44	19	1	11	9	/	19	1
	Min = 29; max = 60							

**Table 2:** Facilitators of self-sampling according to all health professionals.

		Extract/Example
Offset low medical density	Access to care	“They already have trouble accessing care, doctors” MID14
	Delays for appointments	“Women who request an appointment today get it in seven months” Gyn9
	Possibility of screening	“I do not offer gynecological consultation” GP7
Target	Geographic isolation	“There are places where the nearest doctor or midwife is 40 km away” MID18
	Demedicalization	“It simplifies the task and avoids the intrusive and invasive nature of our examinations” GP15 “There are patients who don’t want to be examined by a man” Gyn6
	Comfort	“Self-sampling is private, there’s no one else” GP12, “Self-testing is better for shy women” MID15
	Free of charges	“If it’s reimbursed, people will do it” MID10
Responsibility	Screening reminder	“Try to empower patients about screening (…) the person takes responsibility” GP15

## Data Availability

The data presented in this study are available on request from the corresponding author.

## Appendix A. Semi-Structured Interview Guide for Gynecologists

(1) General data
  - Gender
  - Age
  - Years of practice
  - Urban/Rural
  - Any patients diagnosed with cervical cancer over the past 2 years? If yes, how many?
(2) Screening practice
  - Do you ask all your patients about the Pap smear test?
  - Do you have enough time to explain the importance of screening?
  - What do you think about the shift from a 3-year to a 5-year interval for the screening of women over 30 years? Do you have any fears?
  - If a patient is reluctant to be screened, what do you suggest?
  - Do you do a lot of tracing or just focus on abnormal follow-ups?
  - As part of self-sampling: If the HPV test is positive, do you take an additional sample? If positive, do you perform a colposcopy?
  - Would you go into nursing homes to reach more of the population?
  - How do you see the future of screening?
(3) Factors influencing screening
  - In your opinion, does immigrant status make a difference?
  - A study conducted in Nantes (France) showed that religion could be a factor for misunderstandings regarding screening: What do you think?
  - Are certain characteristics (e.g., obesity) barriers to screening?
  - Which material conditions do you need for the smear test to go well?
  - Which women do you have difficulty with?
  - Are your female patients with chronic long-term illness up to date with their screening?
  - Do you think you are an obstacle to the screening process?
  - Are there women you don’t ask about screening?
  - In your opinion, what can be done to promote a) screening and b) self-sampling?
  - Do you have any ideas about how to increase screening among women who do not come to consult?
(4) Gynecologists on screening
  - What do you think about screening and self-sampling?
  - Do you think screening through self-sampling is different from other types of screening?
  - Do you differentiate between vaginal and urinary self-sampling?
  - Do you think vaginal self-sampling and urinary self-sampling are different?
  - In your opinion, are there specific benefits linked to self-sampling?
(5) Various issues
  - Do any of your patients refuse screening?
  - Do you see any benefits in handing out self-sampling kits? If yes, which ones?
  - Does self-sampling seem relevant to you? If yes, why?
  - Regarding the delivery of self-sampling kits, would it be best to (1) deliver them yourself (2) ask for them to be sent to the regional cancer screening coordination center (3) make them available for pick-up at a pharmacy or 4) at a medical laboratory?
  - Do women who have received a self-sampling kit come to see you? If so, do they come because they don’t understand the kit or do they ask you to do it for them?
  - Regarding the kits: what do women tell you about their use? Do they have any reservations? What is their experience?
  - Do you trust self-sampling? What do you think? Are you aware that you will have to perform a Pap test if the HPV test comes back positive?
  - Would you be confident if the self-sampling recommendations only concerned vaginal self-sampling and not urinary self-sampling?
  - Do you think that self-sampling is easier? In your opinion, is a medical examination of the cervix mandatory?
  - In your opinion, does self-sampling have a future? If yes, which one?

## Appendix B. Semi-Structured Interview Guide for GPs

(1) General data
  - Gender
  - Age
  - Years of practice
  - Urban/Rural
  - Any patients diagnosed with cervical cancer over the past 2 years? If yes, how many?
(2) Screening practice
  - Do you ask all your patients about the Pap smear test?
  - Do you have enough time to explain the importance of screening?
  - Do you think that GPs are sufficiently aware of the importance of screening?
  - Are you interested in gynecology? If not, who do you refer your patients to?
(3) Factors influencing the performance of screening
  - Shyness and therapeutic alliance: Do you sometimes feel embarrassed, leading you to refer your patients to another health professional, for fear of breaking the therapeutic alliance?
  - In your opinion, does immigrant status make a difference?
  - A study conducted in Nantes (France) showed that religion could be a factor for misunderstandings regarding screening: What do you think?
  - Are certain characteristics (e.g., obesity) barriers to screening?
  - Which material conditions do you need for the smear test to go well?
  - Which women do you have difficulty with?
  - Are your female patients with chronic long-term illness up to date with their screening?
  - Do you think you are an obstacle to the screening process?
  - Are there women you don’t ask about screening?
  - In your opinion, what can be done to promote (a) screening and (b) self-sampling?
  - Do you have any ideas about how to increase screening among women who do not come to consult?
(4) GPs on screening
  - What do you think of screening and self-sampling?
  - Do you think self-sampling is different from other types of screening?
  - Do you think vaginal self-sampling and urinary self-sampling are different?
  - In your opinion, are there specific benefits linked to self-sampling?
  - Self-sampling has been recommended since 2019. Since 2020, vaginal self-sampling is reimbursed by the social security system, together with the reminder letter. Do you see any value in these recommendations?
(5) Various issues
  - Do any of your patients refuse any sampling?
  - Do you see any benefits in handing out self-sampling kits? If so, which ones?
  - Does handing out self-sampling kits seem relevant to you? If so, why?
  - Regarding the delivery of self-sampling kits, would it be best to (1) deliver them yourself (2) ask for them to be sent to the regional cancer screening coordination center (3) make them available for pick-up at a pharmacy or (4) at a medical laboratory?
  - Do women who have received a self-sampling kit come to see you? If so, do they come because they don’t understand the kit or because they want you to do it for them?
  - Regarding the kits, what do women tell you about their use? Do they have any reservations? What is their experience?
  - Do you trust self-sampling? What do you think? Are you aware that you will have to perform a Pap test if the HPV test comes back positive?
  - Would you be confident if the self-sampling recommendations only concerned vaginal self-sampling and not urinary self-sampling?
  - Do you think self-sampling is easier? In your opinion, is a medical examination of the cervix mandatory?
  - In your opinion, does self-sampling have a future? If so, which one?

## Appendix C. Semi-Structured Interview Guide for Midwives

(1) General data
  - Gender
  - Age
  - Years of practice
  - Urban/Rural
  - Any patients diagnosed with cervical cancer over the past 2 years? If so, how many?
(2) Screening practice
  - Do you ask all your patients about the Pap smear test?
  - Do you have enough time to explain the importance of screening?
  - Do you think that midwives are sufficiently aware of screening?
  - Do you feel comfortable with screening?
  - Have you been trained in screening?
  - Midwives can perform colposcopies. What do you think?
  - How do you see the future of screening?
(3) Factors influencing the performance of screening
  - Shyness and the therapeutic alliance: Do you sometimes feel embarrassed, leading you to refer your patients to another healthcare professional, for fear of breaking the therapeutic alliance?
  - In your opinion, does immigrant status make a difference?
  - A study conducted in Nantes (France) showed that religion could be a factor for misunderstandings regarding screening: What do you think?
  - Are certain characteristics (e.g., obesity) barriers to screening?
  - Which material conditions do you need for the smear test to go well?
  - Which women do you have difficulty with?
  - Are your female patients with chronic long-term illness up to date with their screening?
  - Do you think you are an obstacle to the screening process?
  - Are there women you don’t ask about screening?
  - In your opinion, what can be done to promote (a) screening and (b) self-sampling?
  - Do you have any ideas about how to increase screening among women who do not come to consult?
(4) Midwives on screening
  - What do you think about screening and self-sampling?
  - Do you think that self-sampling is different from other types of screening?
  - Do you think vaginal self-sampling and urinary self-sampling are different?
  - In your opinion, are there specific benefits linked to self-sampling?
  - Self-sampling has been recommended since 2019. Since 2020, vaginal self-sampling is reimbursed by the social security system, together with the reminder letter. Do you see any value in these recommendations?
(5) Various issues
  - Do any of your patients refuse sampling?
  - Do you see any benefits in providing self-sampling kits? If so, which ones?
  - Does handing out self-sampling kits seem relevant to you? If so, why?
  - Regarding the delivery of self-sampling kits, would it be best to (1) deliver them yourself (2) ask for them to be sent to the regional cancer screening coordination center (3) make them available for pick-up at a pharmacy (4) or at a medical laboratory?
  - Do women who have received a self-sampling kit come and see you? If so, do they come because they don’t understand the kit or because they want you to do it for them?
  - Regarding the kits, what do women tell you about their use? Do they have any reservations? What is their experience?
  - Do you trust self-sampling? What do you think? Are you aware that you will have to perform a Pap test if the HPV test comes back positive?
  - Would you be confident if the self-sampling recommendations only concerned vaginal self-sampling and not urinary self-sampling?
  - Do you find self-sampling easier? In your opinion, is a medical examination of the cervix mandatory?
  - In your opinion, does self-sampling have a future? If so, which one?
